# Targeted metagenomics as a tool to tap into marine natural product diversity for the discovery and production of drug candidates

**DOI:** 10.3389/fmicb.2015.00890

**Published:** 2015-08-28

**Authors:** Marla Trindade, Leonardo Joaquim van Zyl, José Navarro-Fernández, Ahmed Abd Elrazak

**Affiliations:** ^1^Institute for Microbial Biotechnology and Metagenomics, University of the Western Cape, BellvilleSouth Africa; ^2^Centro Regional de Hemodonación, Servicio de Hematología y Oncología Médica, Universidad de Murcia, IMIB-Arrixaca, MurciaSpain; ^3^Botany Department, Faculty of Science, Mansoura University, MansouraEgypt

**Keywords:** uncultured microbes, metagenomics, symbionts, marine natural products, drug discovery, functional screening

## Abstract

Microbial natural products exhibit immense structural diversity and complexity and have captured the attention of researchers for several decades. They have been explored for a wide spectrum of applications, most noteworthy being their prominent role in medicine, and their versatility expands to application as drugs for many diseases. Accessing unexplored environments harboring unique microorganisms is expected to yield novel bioactive metabolites with distinguishing functionalities, which can be supplied to the starved pharmaceutical market. For this purpose the oceans have turned out to be an attractive and productive field. Owing to the enormous biodiversity of marine microorganisms, as well as the growing evidence that many metabolites previously isolated from marine invertebrates and algae are actually produced by their associated bacteria, the interest in marine microorganisms has intensified. Since the majority of the microorganisms are uncultured, metagenomic tools are required to exploit the untapped biochemistry. However, after years of employing metagenomics for marine drug discovery, new drugs are vastly under-represented. While a plethora of natural product biosynthetic genes and clusters are reported, only a minor number of potential therapeutic compounds have resulted through functional metagenomic screening. This review explores specific obstacles that have led to the low success rate. In addition to the typical problems encountered with traditional functional metagenomic-based screens for novel biocatalysts, there are enormous limitations which are particular to drug-like metabolites. We also present how targeted and function-guided strategies, employing modern, and multi-disciplinary approaches have yielded some of the most exciting discoveries attributed to uncultured marine bacteria. These discoveries set the stage for progressing the production of drug candidates from uncultured bacteria for pre-clinical and clinical development.

## Marine Microorganisms as a Novel Source of Natural Products

Natural products remain a major resource for drug production today and during the past 30 years, 70% of antimicrobials and 60% of chemotherapeutics have been developed or analogously synthesized from them (Pomponi, 2001; Grüschow et al., 2011). Traditionally, terrestrial sources have provided the bulk of natural products for drug molecules. However, participation by the major pharmaceutical companies declined in the mid-nineties, largely owing to the high rediscovery rate and decreased number of novel compound identifications (Molinski et al., 2009). In the meantime infectious diseases and multiple drug resistant strains have bloomed, urging scientists to mine for novel drugs in non-terrestrial and unexplored environments. A chemoinformatics study showed that 71% of the marine natural products were not represented in terrestrial natural products, and that 53% have been found only once (Montaser and Luesch, 2011). Complementary studies investigating the distribution of natural products in chemical space has shown clearly that marine natural products have the broadest distribution, covering many drug-relevant areas (Tao et al., 2015). As such, the focus has recently shifted to marine natural product bioprospecting, which has delivered remarkably high hit rates (Gerwick and Moore, 2012; Blunt et al., 2015).

The ocean harbors a number of ecological niches and has proven to be home to more microorganisms than any other environment. Considering that 70% of our planet’s surface is covered by the oceans, it is not surprising that certain marine ecosystems harbor much higher biological and chemical diversity than what is found terrestrially. Furthermore, the sedentary lifestyle of many of the organisms necessitates a chemical means of defense, and as such natural products are produced as chemical weapons which have evolved into highly effective inhibitors (Spainhour, 2005). Since the released compounds become rapidly diluted, marine natural products tend to be highly potent in order to be effective (Haefner, 2003). The rich biodiversity contained within the oceans (15 animal phyla exclusive to the oceans) makes them a unique and rich drug discovery reservoir (Leal et al., 2012).

Marine natural product discovery was initially focused on the easily accessible macro-organisms (such as algae, soft corals, and sponges) from which a range of bioactive compounds have been described (Bergmann and Feeney, 1951; McGivern, 2007; Hu et al., 2011; Leal et al., 2012). However, efforts have gradually turned to the smaller forms of life such as bacteria and fungi (Gerwick and Moore, 2012) which constitute a large portion of the marine biomass (Sogin et al., 2006). Considering the enormous number of microbes, their vast metabolic diversity and the rate of mutations during the past 3.5 billion years, it is expected that there are high levels of genetic and phenotypic variation in marine environments (Sogin et al., 2006). Furthermore, marine microorganisms live in a biologically competitive environment with unique, harsh, and fluctuating conditions. They encounter enormous physical and chemical variability including low temperature, high pressure, oligotrophy, high salinity and other competitive environments, and are especially rich in chlorine and bromine elements. Global scale analyses of bacterial diversity identify environment salinity and temperature as the major determinants of microbial community composition, resulting in distinct marine microbiota being selected (Lozupone and Knight, 2007). Biofilm formation is a crucial aspect where cell densities are typically 100–1000 fold higher in a biofilm assemblage than in the surrounding water column (Wahl et al., 2012). Furthermore, the increased competition amongst organisms is thought to be the source of higher production levels of secondary metabolites (Teasdale et al., 2009). In contrast to typical terrestrial environments, marine environments have a very high bacterial diversity at the higher taxonomic levels and a global biogeographical study has shown that there is no more than 12% taxon overlap between bacterial assemblages within and between habitat types (Nemergut et al., 2011). As a result marine microorganisms represent a unique source of genetic information and biosynthetic capacity which translates to huge chemical diversity.

## Marine Microbial Natural Products

Marine microorganisms produce a vast variety of secondary metabolites which could be used to supply the starved pharmaceutical market. Microbial natural products have notable potent therapeutic activities, and also often possess the desirable pharmacokinetic properties required for clinical development (Farnet and Zazopoulos, 2005). More than half of the known natural products with anti-microbial, anti-tumor (Bewley and Faulkner, 1998; Feling et al., 2003; Taori et al., 2008; Rath et al., 2011) or anti-viral activity are of bacterial origin (Berdy, 2005). Additional categories include anti-parasitic (Kirst et al., 2002; Abdel-Mageed et al., 2010), anti-nematodal (Donia and Hamann, 2003), anti-inflammation (Strangman, 2007), and neurological (Sudek et al., 2007). Pharmaceutically relevant natural products belong to different chemical classes that differ not only in structure, but also in the mechanisms by which they are synthesized. The molecular classes which become pharmaceuticals tend to be alkaloids, terpenoids, polyketides and small peptides, and a wide range of bioactive properties are observed within each class (Graça et al., 2013). Furthermore, the elucidation of novel hybrid compounds is providing deeper insights into fascinating enzyme assemblies and mechanisms behind the diversity in structure and biological functions observed in these compounds. Some marine derived microbial examples can be found in the following references: alkaloids (Charan et al., 2004; Abdelmohsen et al., 2012); terpenoids (Kuzuyama and Seto, 2003; Cho et al., 2006; Strangman, 2007; Solanki et al., 2008); polyketides (Olano et al., 2009; Harunari et al., 2014), peptides (Pettit et al., 2009; Chopra et al., 2014); and hybrids (Hardt et al., 2000; Feling et al., 2003; Oh et al., 2007; Blunt et al., 2015).

An additional attraction of microbially derived natural products is that they offer an answer to the supply problem, a major bottleneck in the drug discovery pipeline. The progression of many marine natural products with promising pharmaceutical relevance into clinical phases are halted since the clinical trial stage requires a considerable amount of drug mass; usually kilogram amounts (Tsukimoto et al., 2011). Most pharmaceutically interesting compounds are found in minute amounts, therefore bioprospecting cannot rely on wild-harvesting as it could lead to the extinction of marine species. More economically feasible, environmentally friendly, and sustainable sources of lead compounds are required. Microbial-based production of lead compounds therefore offers a sustainable solution through the use of culturable marine microorganisms (microbial fermentation). Marine bacteria can respond positively during scaling up processes, and can incorporate sustainable chemical processes for faster establishment of a pilot plant for production (Abd Elrazak et al., 2013). The current industrial process for the production of Yondelis, for example, involves the fermentation of *Pseudomonas fluorescens* for the production of the starting material cyanosafracin B, followed by semi-synthesis to generate the final drug (Cuevas et al., 2000). Furthermore, strain intensification and elicitation to improve expression are possible through metabolic engineering, as well as the unlocking of untapped cryptic biosynthetic pathways through heterologous host expression (Li and Neubauer, 2014).

## Marine Metagenomics

There is remarkable potential harbored within microorganisms to produce diverse drug-like small molecules for a wide range of applications. The impact and possible success of a single new discovery distinguishes natural products from all other sources of chemical diversity (Farnet and Zazopoulos, 2005). However, traditional culture-based approaches used to identify microbial metabolites likely miss the vast majority of bacterial natural products. Only about 1% of bacteria are cultured *in vitro* and of the approximately 61 bacterial phyla known, 31 lack cultivable representatives (Vartoukian et al., 2010). Seawater bacteria have a 10-fold lower representation of cultured isolates compared to other environments (Amann et al., 1995). Therefore, if the natural products discovered from cultured marine bacteria are an indication of the diversity available, culture-independent approaches are expected to more successfully access the untapped reservoir of chemical diversity and contribute many more novel marine-derived discoveries.

The study of DNA obtained directly from an environmental sample (metagenomics) accesses the collective genomes and bioactive potential of bacterial consortia (Handelsman, 2004). Metagenomics therefore provides a means of exploring novel metabolites from bacteria that are known to be present in marine environments but which remain recalcitrant to culturing (Banik and Brady, 2010). Moreover, metagenomics is particularly attractive for natural product discovery because the genetic information encoding the activities of interest are generally clustered on bacterial genomes, making it possible to clone an entire pathway on an individual or at least a small number of overlapping library clones (Handelsman et al., 1998; Banik and Brady, 2010). Therefore, high throughput metagenomic screening approaches, using both sequence-based and function-based screening, can be employed, in theory, to de-replicate known pathways and compounds and reduce the high degree of redundancy obtained through traditional culture based approaches. Metagenomic screening approaches cover a large range of techniques and are subject to the specifications of the target compound. The particular focus of this review is to evaluate the impact of function-guided strategies as a tool in marine natural product discovery. Specifically, we compare two different functional approaches and their contributions to unlocking the natural product potential harbored in marine microbial genomes.

## Classic Functional Metagenomic Screening

**FIGURE 1:**
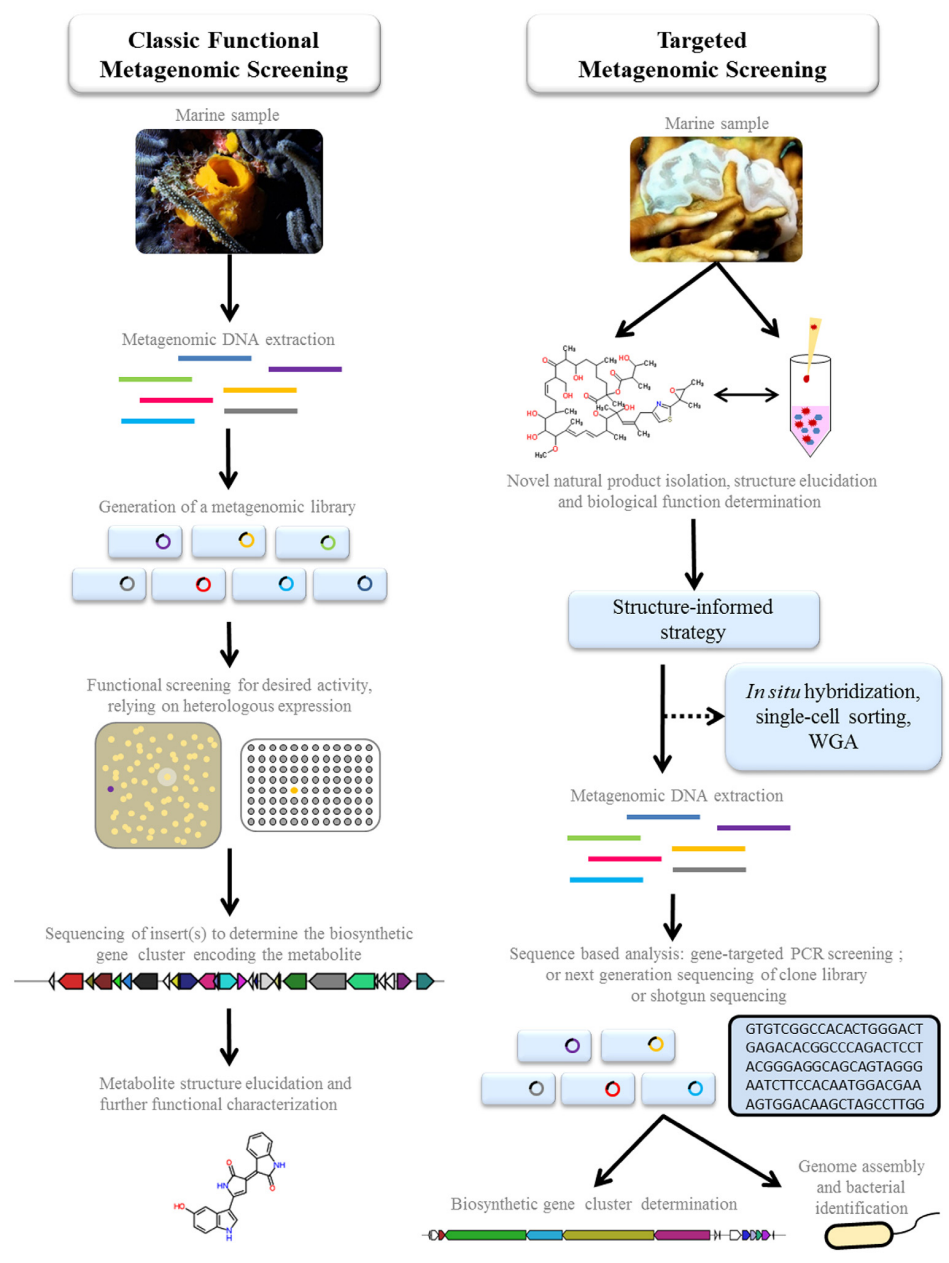
**A comparison of two function-driven approaches to employ metagenomics for the discovery and production of pharmaceutically relevant marine natural products.Classic functional metagenomic screening:** metagenomic libraries are generated in a suitable host and activity screened in a variety of ways, to detect clones expressing metabolites with potential therapeutic properties. The active clones are sequenced to determine the biosynthetic pathway. For certain classes of secondary metabolites, sequence from overlapping clones may be required to compose the entire pathway. The structure of the expressed metabolite is elucidated, following chemical dereplication and characterization methods. If the metabolite is novel, further functional characterization is conducted to evaluate its therapeutic potential. **Targeted metagenomic screening**: these strategies are guided by traditional chemistry and structure/function-based discoveries in which novel natural products are first isolated and characterized directly from the marine organism or environment. Guided by the chemical classification, a targeted sequence-based analysis can be employed to identify whether the metabolite is microbially encoded, and to subsequently describe the biosynthetic gene cluster. This approach has been employed successfully (detailed in text) when integrated with a number of technologies such as *in situ* hybridization, single-cell sorting, and whole genome amplification (WGA). The sequence-based analysis of the metagenomic DNA can include gene-targeting using degenerate PCR amplification; or next generation sequencing of the clone library or of the metagenomic DNA directly (shotgun). Where sufficient sequence information is assembled, full genome information can be used to describe novel and uncultured bacteria. The elucidation of the genetic clusters provides the foundation for direct production of the pharmaceutical drug and new analogs through metabolic engineering, and opens the possibility to produce the drugs through heterologous expression.

In natural product discovery, classical functional screening involves the generation and subsequent screening of metagenomic libraries for the direct detection of the metabolite’s properties (e.g., antibacterial, antifungal, antitumor, antiviral activity; Rocha-Martin et al., 2014; **Figure 1**). Using whole cells, the culture supernatant or cell pellet extract, this screening approach has been employed with some success. One of the simplest strategies is to test for growth inhibition against a test microbe in top agar overlay assays. This has led to the characterization of a variety of new antibiotics from soil-derived environments (Brady and Clardy, 2000, 2005; Curtois et al., 2003), but no marine-derived studies have been reported, to our knowledge. A more typical approach to functional screening is to screen for a readily detectable phenotype which is representative of the desired bioactive compound, either through the visual detection of pigment production or the use of chromogenic and fluorogenic enzyme substrates which allow the detection of specific catalytic functions encoded on individual clones when incorporated into the growth medium (Ferrer et al., 2009; Guazzaroni et al., 2015). The antibacterial pigments violacein (Brady et al., 2001), indigo (Lim et al., 2005), and turbomycins (Gillespie et al., 2002) have been isolated from soil metagenomic libraries. Success with marine libraries; however, has not been reported. A number of other function based screens have yielded a range of different bioactive compounds or activities. Although these screens have not been employed in marine library screening, they are worthy of mention because we expect it is only a matter of time before they are reported. An acylhomoserine lactone synthase promoter fused to a *lacZ* reporter has been employed to identify AHL lactonases capable of inhibiting *Pseudomonas aeruginosa* biofilms (Schipper et al., 2009). A phosphopantetheinyl transferase (PPTase)-targeting functional screen has resulted in the efficient recovery of natural product gene clusters from metagenomic libraries (Owen et al., 2012). Non-ribosomal peptide synthetase and polyketide synthase (PKS) enzymes are activated by PPTases, therefore these enzymes are frequently associated with secondary metabolite gene clusters (Osbourn, 2010; Owen et al., 2012). There is a much greater chance of detecting the expression of a single intact gene than an entire biosynthetic operon, therefore focusing on only a single gene target for the recovery of NRPS and PKS gene clusters by association increases the chances of identifying “hits” (Owen et al., 2012).

Function-driven screening strategies offer significant advantages to sequence/homology based screening (Tuffin et al., 2009; Kennedy et al., 2010; Suenaga, 2012). This is primarily due to the fact that prior knowledge of the gene sequence for the target activity of interest is not needed, and as a result it is expected that functional screening increases the ‘novelty’ hit rate. This increases the potential of identifying entirely new classes of genes for both known and novel functions (Sharma and Vakhlu, 2014). Furthermore, the hits obtained represent an “insurance policy”; guaranteed success of expression in the heterologous host, enabling one to screen for particular properties and under specified conditions, as well as facilitating downstream analyses. The dearth of marine natural product discoveries through functional metagenomics is puzzling considering the increased research focus on marine microorganisms over the last decade (Kennedy et al., 2010). We propose two major reasons for this, (i) heterologous expression challenges and (ii) the sequence technology boom.

## Challenges Associated with Classic Functional Metagenomics

Natural product discovery, using metagenomics, faces a number of significant challenges and limitations when employing classic functional screening approaches (Kennedy et al., 2010; Li and Neubauer, 2014; Reen et al., 2015). The most well-known are those associated with heterologous gene expression. Gabor et al. (2004) estimated using *in silico* analysis that only 40% of enzymatic activities can be identified by random cloning of environmental DNA in *Escherichia coli*. Many studies have highlighted heterologous expression as an enormous challenge limiting the robustness of metagenomics to fully access metabolic potential (Ferrer et al., 2009; Uchiyama and Miyazaki, 2009; Reen et al., 2015). In natural product discovery, these challenges are augmented for a number of reasons.

(i) Unlike for other biotechnologically important enzymes and activities typically screened in metagenomic studies, such as the glycosyl hydrolases for example, the activities encoded by particularly the PKS and NRPS pathways, require optimal induction conditions of many genes for expression. The enzymatic megacomplexes for dedicated synthesis of their cognate products are encoded by massive gene clusters, some composed of over 20 genes which are distributed between multiple polycistronic transcriptional units (Gao et al., 2010; Osbourn, 2010). Obviously there is a much lower chance of expressing an entire biosynthetic pathway in any given heterologous host than a single active enzyme. Secondary metabolite pathways are regulated by pathway specific proteins as well as global regulatory elements in response to changes in nutrient conditions or environmental signals (Van Wezel and McDowall, 2011). The extremely diverse marine specific factors responsible for unique biochemistries are difficult to replicate in functional screening. For example, it is well-understood that many secondary metabolite pathways expressed in their natural environmental conditions remain silent under laboratory conditions (Montaser and Luesch, 2011), and this is magnified in heterologous systems. The synergies associated with complex symbiotic and competitive interactions cannot easily be incorporated in simple expression systems.

(ii) Even if heterologous expression of a particular pathway is successful, it may not necessarily produce the same compound. Only one isomer may be active and not the other due to the requirement of intermediate compound(s) from the original host or environment (Taylor et al., 2007; Sagar et al., 2010). Furthermore, the absence of a required post-translational modification process, the requirement of *in trans* genetic elements or the fragmentation of previously clustered genes would not allow functional detection (Kwan et al., 2012; Nakabachi et al., 2013). The use and development of alternative bacterial hosts, expression systems, and multi-host shuttle vectors is crucial to overcoming the limitations discussed. The ability to screen using alternative transcriptional machinery and promoter recognition capabilities should broaden the spectrum of gene expression. Recently, in order to achieve good heterologous expression of novel bioactive compounds, the development of marine-derived hosts such as actinomycete, cyanobacteria, and symbiotic fungi was undertaken to optimize heterologous production (Rocha-Martin et al., 2014). The ability to replicate in multiple hosts enables the screening to be conducted in the background of different regulatory and metabolic networks. Furthermore, biosynthetic pathways have also been shown to result in different phenotypes when expressed in different hosts (Craig et al., 2010).

(iii) Owing to the large sizes of the biosynthetic pathways, which routinely approach 100 kb, functional screening of metagenomic libraries for the encoded activity is restricted by the need for the entire cluster to be recovered on a single clone (Kim et al., 2010). Libraries therefore need to be prepared in bacterial artificial chromosomes (BACs), which can be maintained at low copy number and can carry DNA inserts as large as 350 kb (Shizuya and Simon, 1992). However, it is a major technical challenge to preserve the large size of the metagenomic DNA while sufficiently removing impurities that inhibit cloning. In practice, metagenomic BAC libraries only manage 40 kb insert sizes and rarely greater than 70–100 kb (Handelsman et al., 1998; Kakirde et al., 2010). Furthermore, metagenomes representing symbiotic communities associated with marine invertebrates represent hundreds of individual genomes. To adequately represent each one requires massive DNA libraries, in the order of 10^6^ clones, to be constructed and screened (Freeman et al., 2012). Therefore, metagenome libraries generally vastly underrepresent the true diversity, which has so far prohibited the realization of a functional metagenomic approach (Fisch et al., 2009).

(iv) Activities which are initially identified and associated with a library clone extract are sometimes lost before the chemical structure can be determined due to strong negative selection in the heterologous system (Curtois et al., 2003).

(v) Microbial-derived compounds often have multiple activities; for example anti-tumor (Abbas et al., 2013; Du et al., 2013), anti-inflammatory (Chandak et al., 2014), and anti-protozoan (Abdel-Mageed et al., 2010) compounds also display antibacterial activity which may be toxic to the heterologous host. A large proportion of sought-after activities will therefore never be represented in metagenomic libraries. This cannot necessarily be overcome by the use of shuttle vectors because it is in the initial library construction phase that the clones harboring toxic activities will be lost. Ideally metagenomic libraries constructed in shuttle vectors need to be transformed/transfected into the multiple hosts; however, the levels of efficiency required are difficult to generate in non-*E. coli* hosts. Maintaining low copy numbers may enable the host to survive the toxicity; however, it is highly likely that the screening method will not be sensitive enough to detect the active clone.

## The Sequence Boom

To overcome some of the challenges associated with functional screening, sequence/homology-based screening has been employed in a number of different ways. It is not the intention of this review to compare function vs. sequence based metagenomic methods; however, a brief review is presented to put into context the need for continued attention to functional metagenomic tools.

Metagenomic DNA or clone libraries can be screened using degenerate PCR primers designed to conserved sequences within biosynthetic gene clusters (Banik and Brady, 2010). The clustering of biosynthetic genes on a contiguous region of DNA makes homology-based screening attractive. The domain architecture of PKSs and NRPSs in most cases mirrors the structure of the assembled metabolite (Piel et al., 2004c). Therefore, the use of degenerate primers is routinely and successfully employed to first detect conserved NRPS and PKS motifs, followed by the recovery of the remainder of the biosynthetic enzymes by association (Moffitt and Neilan, 2001; Dunlap et al., 2007; Bayer et al., 2013). Furthermore, the identification of relatives of known biosynthetic variants could be a strategy to identify or synthesize new structural variants to provide compounds with improved pharmacological properties (Banik and Brady, 2010). However, in some cases up to 99% of the genes detected through PCR screening can represent dominant sequences which are already known and alternative strategies are required to overcome the presence of similar sequences (Piel et al., 2004c; Schirmer et al., 2005; Fieseler et al., 2007; Kennedy et al., 2008; Hochmuth et al., 2010; Siegl and Hentschel, 2010; Pimentel-Elardo et al., 2012; Della Sala et al., 2013, 2014).

Exciting advancements in next generation DNA sequencing and bioinformatics technologies now negates the need to prepare and sequence clone-libraries. Shotgun metagenomic sequencing has made it possible to rapidly identify large biosynthetic gene clusters and subsequent predictions of their chemical structure can be made (Caboche et al., 2008, 2010; Röttig et al., 2011; Medema et al., 2012, 2014; Blin et al., 2013). While purely *in silico* approaches are generally limited to the detection of one or more well-characterized gene cluster classes (Cimermancic et al., 2014), continued developments in bioinformatics pipelines and other technologies are already improving access to diverse and novel secondary metabolite genes and clusters, including providing access to the “rare biosphere” (we refer readers to a number of examples: Li et al., 2010; Sagar et al., 2010; Trindade-Silva et al., 2012; Woodhouse et al., 2013; Cimermancic et al., 2014). Furthermore, the deposition of more functionally curated sequence data in publically available databases should improve the ability to use purely bioinformatics based screening for the identification of novel gene clusters (Tuffin et al., 2009; Suenaga, 2012).

*In silico* approaches facilitate rapid dereplication of common biosynthesis clusters and thus the prioritization of new chemical scaffolds for experimental characterization. Although, targeted induction in heterologous expression systems has delivered some success from the marine environment (Long et al., 2005; Schmidt et al., 2005; Hochmuth et al., 2010; Rath et al., 2011; Bonet et al., 2015; Li et al., 2015), it is not easily going to deliver compounds with the sought after properties required by the pharmaceutical markets in a high throughput manner, when taking a purely *in silico* discovery route. For example, the *swf* cluster, a new mono-modular type I PKS/FAS (fatty acid synthase) was identified through screening of the *Plakortis simplex* sponge metagenome (Della Sala et al., 2013). The entire pathway was expressed in *E. coli*; however, the production of an associated metabolite was not detected.

Notwithstanding all the difficulties associated with heterologous expression and the inability to conduct this in a high throughput manner, novel sequence will not necessarily result in the pharmaceutically required biological properties. It is currently easier and cheaper to generate vast volumes of gene and genome sequence information than it is to produce the experimental characterizations, and the gap between these is growing rapidly (Prakash and Taylor, 2012; Scholz et al., 2012; Teeling and Glöckner, 2012; Reen et al., 2015).

## Targeted Metagenomic Strategies in Marine Discovery

From a pharmaceutical point of view, marine drug discovery necessitates a focus on functionality. Irrespective of the approach employed, obtaining biologically active and pure compounds with the desired activity or properties is the end goal. The ability to achieve this through function-driven screening strategies is, in principle, the golden standard. Given the limitations discussed above this will remain a major challenge. Relative to other environmental biodiscovery efforts, classical functional metagenomic screening of marine sources has yet to contribute significantly to the pharmaceutical industry. However, significant improvements in the chemical and genetic sciences and the integration of these technologies, has resulted in a number of successes which are beginning to drive the development of parallel technologies.

Instead of functionally screening a metagenome clone library, a targeted approach which harnesses prior knowledge of marine natural product diversity, chemistry, and biological activity is bridging the gap between the accumulation of microbial genetic datasets and functional and ecological relevance (**Figure 1**). In this section we highlight some of the recent discoveries that have employed metagenomic strategies which were guided primarily by initial structural and functional characteristics and associated pharmaceutical interest.

### Bryostatins

Bryostatin 1, a polyketide initially detected in 1968 in extracts from the marine bryozoan *Bugula neritina* (Pettit, 1991), raised interest due to its cytotoxic activity against multiple carcinomas, with proteinase kinase C as its molecular target (Mayer et al., 2010). Bryostatin 1 has been tested in over 80 clinical trials for cancer and is also being assessed in Phase I trials as an anti-Alzheimer’s drug. Although the *in vivo* activity was initially detected directly from the bryozoan, it was for many years suspected that the compound was produced by a bacterial symbiont since a difference in the types of bryostatins found in *B. neritina* correlated with genetically different bacterial symbionts (Davidson and Haygood, 1999). A particular symbiont in the larvae of the bryozoan was identified and suspected to be the producer, and was proposed as a novel gamma-proteobacterium, ‘*Candidatus* Endobugula sertula.’ Attempts to separate the bacterial cells from the host as a way to confirm *Ca* E. sertula as the producer of the bryostatin were inconclusive, therefore a metagenomic approach was employed (Davidson et al., 2001). Since, bryostatin is a type I polyketide, PKS-based screening was conducted and led to the confirmation that the genes coding for type I PKS complex were derived from the symbiotic population. Further query involving the growth of *B. neritina* colonies after antibiotic treatments and *in situ* hybridization experiments confirmed that “*E. sertula*” was the source of the bryostatins. A cosmid library was prepared from *B. neritina* Mission Bay metagenomic DNA, and was screened by hybridization (Hildebrand et al., 2004) using a β-ketoacyl synthase probe identified previously (Davidson et al., 2001). Several overlapping clones were sequenced revealing the 65 kb *bry* gene cluster (Hildebrand et al., 2004). Probes spanning the *bry* gene cluster were hybridized to ‘*Candidatus* E. sertula’-enriched DNA to confirm the symbiont as the origin of the gene cluster. Further interrogation in two closely related “*E. sertula*” strains from different host species identified two different gene cluster arrangements (Sudek et al., 2007). In one strain the gene cluster is contiguous, while in the other strain the PKS genes are split from the accessory genes. Due to the difficulties in obtaining sufficient supply of the bryostatins, their clinical application occurred decades after their discovery. Since “*E. sertula*” remains unculturable, heterologous expression of the *bry* gene cluster could be considered for the production of bryostatins in large enough quantities for pharmaceutical development.

### ET-743 (Yondelis^®^)

Anti-cancer activity in extract from the sea squirt *Ecteinascidia turbinata* was identified in 1969; however, it was only in 1984 that the structure of one of the compounds, Ecteinascidin 743 (ET-743), was determined (Rinehart, 2000). ET-743 (Yondelis^®^) is now an approved anti-cancer agent (Bewley and Faulkner, 1998). Attempts to farm the sea squirt to provide sufficient supply of the compound had limited success, and it is currently generated in suitable quantities for clinical use by a lengthy semi-synthetic process (Cuevas et al., 2000; Rath et al., 2011). The similarity of ET-743 to three other bacterial derived natural products (saframycin A, *Streptomyces lavendulae*; saframycin Mx1, *Myxococcus xanthus*; safracin B, *Pseudomonas fluorescens*; Rath et al., 2011) suggested that ET-743 was produced by a marine bacterial symbiont. Using metagenomic sequencing of total DNA from the microbial consortium associated with the tunicate resulted in the assembly of a 35 kb contig containing 25 genes encoding a NRPS biosynthetic pathway. Rigorous sequence analysis of two large unlinked contigs suggested that ‘*Candidatus* Endoecteinascidia frumentensis’ was the producer of the metabolite. Subsequent metaproteomic analysis confirmed expression of three key biosynthetic proteins. The complete genome of ‘*Candidatus* Endoecteinascidia frumentensis’ was very recently determined, showing an extremely reduced genome (~631 kb) and evidence of an endosymbiotic lifestyle (Schofield et al., 2015). Having the pathway elucidated provides the foundation for direct production of the drug and new analogs through metabolic engineering (Rath et al., 2011).

### Patellazoles

The *Lissoclinum patella* tunicate has garnered interest due to it representing a rich source of potential bioactive drug leads (Kwan et al., 2012; Schmidt et al., 2012). The patellazoles were isolated directly from the tunicate in the late 1980s and characterized as a new family of novel thiazole-containing polyketide metabolites (Corley et al., 1988; Zabriskie et al., 1988). In addition to their chemical novelty, they gained interest due to their potent cytotoxic activity against human cell lines as well as antifungal (*Candida albicans*) activity (Zabriskie et al., 1988). The patellamides, also isolated from this tunicate had already been shown to be produced by the cyanobacterial symbiont, *Prochloron didemni* (Schmidt et al., 2005). Although, *P. didemni* is the major symbiont, *L. patella* harbors a complex microbiome (Donia et al., 2011), and therefore there stood the possibility that the patellazoles were also produced by a symbiont. Due to the multiple acetate units, patellazoles were hypothesized to be produced by a type I PKS pathway, as well as a NRPS module for the incorporation and cyclization of a cysteine unit to generate the thiazole ring (Kwan et al., 2012). Based on this information an exhaustive sequence based screening of a metagenome clone library prepared from the tunic-cloaca habitats was undertaken, but did not locate the biosynthetic pathway. PCR amplification revealed PKS genes from the *trans-*acyltransferase family, consistent with patellazole biosynthesis, in the tiny zooids but not in the bulk tunic. DNA extracted from the zooids fraction was subjected to shotgun sequencing and the assembly thereof resulted in a complete genome which contained a 86 kb *trans-*AT PKS pathway. The predicted biosynthetic model of the encoded pathway was consistent with patellazoles structure, thus strongly supporting the assignment. The assembled genome was considered to belong to an uncultured symbiont, designated as *Candidatus* “Endolissoclinum faulkneri,” most closely related to free-living marine α-proteobacteria.

### Pederin-led Discovery of the Onnamides

**FIGURE 2:**
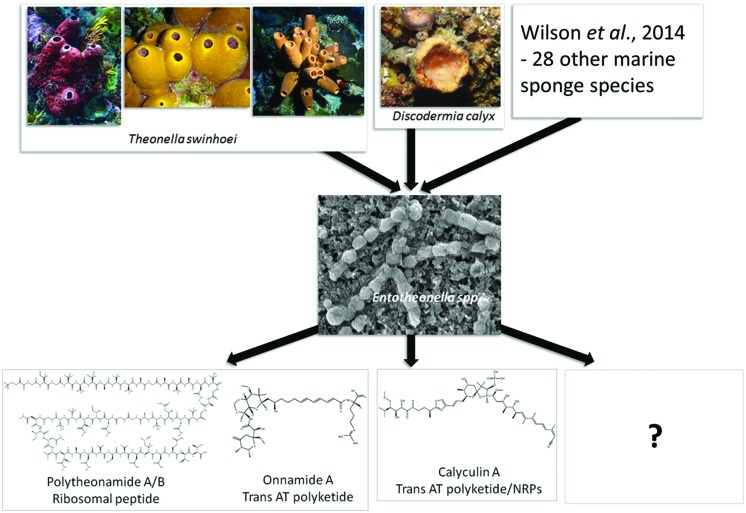
**A representation of the ubiquity of “*Entotheonella*” species in taxonomically diverse marine sponges and the notable secondary metabolites they produce**. Metabolite structure and function information obtained directly from the *Theonella swinhoei* and *Discodermia calyz* sponges informed a targeted metagenomic approach to identify the biosynthetic pathways encoding these metabolites. This ultimately led to the discovery of “*Entotheonella*,” described as “talented producers” due to their chemically diverse metabolism. The full potential of *Entotheonella* species has yet to be explored. Photos were provided by T. Mori, P. Poppe, and T. Wakimoto.

Pederin and mycalamides A and B, encoded by a mixed modular PKS-non-ribosomal peptide synthetase (NRPS) system, are highly active antitumor compounds (Narquizian and Kocienski, 2000). These compounds block mitosis at levels as low as 1 ng/ml by inhibiting protein and DNA synthesis without affecting RNA synthesis (Singh and Yousuf Ali, 2007). They prevent cell division, and have been shown to extend the life of cancerous mice. Consequently they have garnered interest as potential anti-cancer treatments (Kanamitsu and Frank, 1987). These compounds were initially known exclusively from terrestrial *Paederus* and *Paederidus* beetles and after many years of speculation were finally shown to be produced by an uncultured symbiotic *Pseudomonas* associated with the *Paederus fuscipes* beetles using metagenomic approaches (Piel et al., 2004a). Interestingly, these insects use pederin as a chemical weapon against predators and when in contact with human skin cause severe dermatitis (Borroni et al., 1991; Piel et al., 2004a). Metabolites with high structural similarity to pederin were identified in the marine sponges of the order *Lithistida* (Bewley and Faulkner, 1998), many of which exhibit extremely potent antitumor activity and also selectivity against solid tumor cell lines (Cichewicz et al., 2004). A pederin-informed survey of PKS amplicons from the Japanese sponge *Theonella swinhoei* metagenome, a species with exceptionally rich chemistry (Fusetani and Matsunaga, 1993; Bewley and Faulkner, 1998), revealed a wide range of distinct KS sequences (Piel et al., 2004b). Three of these belonged to an evolutionarily distinct enzyme family, the *trans*-acyl-transferase (*trans*-AT) PKSs, and corresponded to onnamide biosynthesis pathways. These *trans*-AT PKSs therefore were expected to encode the pederin-like compounds with antitumor activity produced by the sponges. Further, screening of the metagenomes from other *T. swinhoei* specimens revealed that these *trans*-AT PKSs could only be detected in the sponges which had previously shown to contain pederin-type compounds, while no amplification was obtained for sponges devoid of these compounds. It has now been confirmed that the onnamides (**Figure 2**) are produced by an unculturable symbiont, ‘*Candidatus* Entotheonella spp.’ (Wilson et al., 2014).

### Psymberin

Psymberin, a highly cytotoxic and selective antitumor polyketide, has been isolated from a number of different marine sponges (Bielitza and Pietruszka, 2013). It took 11 years and 600 samples for the structure of this compound to be assigned. There is immense interest in this natural product due to its complex architecture, biological properties and scarcity in nature. As with the onnamides, psymberin is a member of the pederin family, also synthesized by a *trans*-AT PKS (Piel et al., 2004b), but is distinguished from the other pederins due to its excellent cytotoxicity values which exceeds those of the other family members. A *trans*-AT PKS PCR screening approach, as described above for the onnamides, was used to elucidate the complete biosynthetic pathway for psymberin from the *Psammocinia aff. bulbosa* sponge metagenome (Fisch et al., 2009). The genomic region showed typical bacterial architecture, suggesting a bacterial symbiont origin. However, unlike for the pederin and onnamide examples, there were not enough similarities to other bacterial genes to identify the bacterium.

### Polytheonamides

The polytheonamides (**Figure 2**) represent another group of exceptionally potent natural product toxins isolated from the *Theonella swinhoei* sponges, and are particularly noteworthy for their size and structural complexity (Hamada et al., 2010). These 48-residue peptides were expected to be products of a non-ribosomal peptide synthetase, since of the 19 different amino acids that constitute these peptides, 13 are non-proteinogenic. Furthermore, the peptides include multiple D-configured and C-methylated residues. However, the size of the NRP biosynthetic machinery required to produce a 48 residue peptide prompted a search for an unusual ribosomal pathway. With the knowledge of the peptide sequence, degenerate PCR primers were designed to the proposed precursor peptide, and used to conduct a semi-nested PCR from a *T. swinhoei* metagenome (Freeman et al., 2012). Sequenced amplicons revealed codon sequences that precisely corresponded to an unprocessed polytheonamide precursor, not only confirming a ribosomal origin, but also suggesting that it is produced by a bacterial endosymbiont. Further screening of the metagenome library revealed the entire 12 gene biosynthetic pathway. Microscopic analysis of *T. swinhoei* (Y chemotype) samples identified a highly enriched population of fluorescent filamentous bacteria showing morphological similarity to the symbiont ‘*Candidatus* Entotheonella palauensis,’ the suspected producer of antifungal peptides isolated from a Palauan *T. swinhoei* chemotype (Schmidt et al., 2000). Using single cell genomics (fluorescence assisted cell sorting and whole genome amplification) combined with pathway specific PCR, the identification of the polytheonamide producer was attributed to an uncultured “*Entotheonella*” spp. (Wilson et al., 2014). Further, screening using onnamide pathway specific markers indicated that the “*Entotheonella*” spp. were the source of both the onnamide and polytheonamide compounds.

### Calyculin A

Calyculin A was originally described in 1986 as a major cytotoxic compound isolated from *Discodermia calyx*, a marine sponge of the Theonellidae family (Kato et al., 1986), and is to date associated exclusively with marine sponges (Wakimoto et al., 2014). Calyculin A represents a fairly sophisticated and unique structure whose biosynthesis was reminiscent of a polyketide and non-ribosomal peptide hybrid pathway incorporating some remarkable modification processes. Calyculin-related compounds have been isolated from a number of different sponges which hinted toward a symbiont being responsible for its production (Dumdei et al., 1997; Edrada et al., 2002; Kehraus et al., 2002). However, it was only very recently that the biosynthetic gene cluster was identified through a metagenomic approach. Based on the initial hypothesis that calyculin A was a type I polyketide, a metagenome library of *D. calyx* was sequentially screened by PCR amplification using *trans*-AT-type KS, adenylation domain (NRPS) and HMGS-like motif primers (Wakimoto et al., 2014). Spanning over 150 kb, a gene cluster containing 29 KS and 5 A domains was identified. The collinearity between the order of the modules and the order of the biosynthetic reactions provided strong evidence that the cluster encoded calyculin A synthesis. Using the entire gene cluster as a probe and employing CARD-FISH, as well as laser microdissection, a filamentous bacterium was identified to harbor the calyculin pathway. The 16S rRNA sequence of this bacterium displayed 97% identity to the ‘*Candidatus* Entotheonella factor’ isolated from the *T. swinhoei* sponges.

## From Function to Genes to Species

The power of metagenomics to identify novel and pharmaceutically relevant organisms, resulting from first obtaining functional and structural data, has been elegantly represented in the examples discussed above. To demonstrate this further, the “*Entotheonella*” and the ‘*Candidatus* Endolissoclinum faulkneri’ stories are elaborated (**Figure 2**).

Genome sequencing of several of the single cell sorted events in the Wilson et al. (2014) study indicated the presence of two closely related “*Entotheonella*” variants, with 97.6% identical 16S rRNA sequences, and 97% identity to *E. palauensis*. These are only 82% identical to representatives from known bacterial phyla and form a well-separated clade, and therefore have been proposed to represent a new candidate phylum “Tectomicrobia.” Both genomes exceed 9 Mb, representing some of the largest known prokaryote genomes. Analysis of the metabolic genes identified over 28 biosynthetic clusters, encoding ribosomal, polyketide and non-ribosomal peptide biosynthesis. Using bioinformatics based predictions, several of the clusters could be assigned to known bioactive peptides isolated from the Japanese *T. swinhoei*, and together with tandem mass spectrometry-based molecular networking a high diversity of previously unknown metabolite families were identified. The combination of these properties is so rare that the new phylum to which these isolates have been assigned is considered the successor to the Actinobacteria, the well-known source of the majority of the world’s antibiotics and anticancer agents (Jaspars and Challis, 2014). Screening for the distribution of these talented producers indicated that they are geographically widespread and are symbiotically associated with other sponge types (Wilson et al., 2014; **Figure 2**). The discovery of a calyculin producing “*Entotheonella*” symbiotically associated with *D. calyx* further expands the number of biosynthetic enzymes and chemical scaffolds encompassed by this genus (Wakimoto et al., 2014), but also serves to highlight the differences between the “*Entotheonella*” populations in different sponges. Attempts to culture the producing symbionts have been unsuccessful. Access to the genome sequences should give important insights to the organism’s metabolism, and such clues to their physiology could inform on the development of appropriate culturing strategies. Several uncultured symbionts have been successfully isolated using such genome sequence-guided strategies (Renesto et al., 2003; Bomar et al., 2011).

In the Kwan et al. (2012) study the patellazole encoding *Ca.* E. faulkneri genome assembled to a mere 1.48 Mbp and showed extensive genome reduction characteristics. Unlike other bacteria with streamlined genomes, *Ca.* E. faulkneri has distinguishing features which strongly suggest that it could not exist independently of its host, *L. patella*. Phylogenetic analysis of patellazole-containing and patellazole-negative tunicates provides evidence that the symbiont has coevolved with the tunicate host and would therefore be transmitted vertically. The patellazole pathway is the only secondary metabolite pathway encoded in the *Ca.* E. faulkneri genome, and represents >10% of the coding sequence. The maintenance of such a large pathway in a genome that is so streamlined as to eliminate most functions indicates its importance to the symbiotic relationship. However, the patellazoles are highly toxic to eukaryotic cells and are found in high amounts in *L. patella*, and it is intriguing that the host has apparently adapted to tolerate such high concentrations. Clearly the patellazoles provide important chemical defense to the host which in turn ensures that the symbiont is maintained. Interestingly, *Ca.* E. faulkneri is found sporadically in *L. patella* tunicates. Patellazole-positive and negative *L. patella* collected within 250 m of each other show that *Ca.* E. faulkneri is only associated with the patellazole-positive colonies, and only in the zooids fraction. This is despite patellazole-positive and negative colonies having nearly identical tunicate phylogeny, and containing virtually identical microbial communities, with the exception of the *Ca.* E. faulkneri. The exclusive localization of *Ca.* E. faulkneri in the zooids and only in certain *L. patella* colonies is intriguing. Considering the *L. patella* zooids filter feed and excrete waste into the cloacal cavities, this could perhaps provide some leads of investigation to further understanding the *Ca.* E. faulkneri localization and the symbiotic relationship.

These discoveries raise several fundamental biological questions relating to: symbiont and secondary metabolite evolution, mechanisms of natural product symbiosis, the role of the natural products in imparting a direct competitive advantage to individual members of a bacterial consortium, and how these symbiotic interactions contribute to the ecology of the marine environment. However, what is now clearly appreciated is that the genomes of previously uncultured bacteria harbor an unprecedented richness of novel compound diversity, and await discovery.

## Conclusion and Future Prospects

The remarkable exploration of marine organisms and their structurally diverse natural products spans a highly active period of over 40 years (Gerwick and Moore, 2012). With attention turning to marine microorganisms as a source of new natural product chemistry, and the realization that many compounds previously isolated are metabolic products of unculturable microbes, marine metagenomics promises to illuminate new bioactivities and chemistries that were previously unattainable. Despite metagenomics being a relatively young technology, it is globally appreciated that major advances are needed given the challenges that now bottleneck future developments, irrespective of whether functional or sequence guided approaches are to be employed. In order to maximize our ability to harvest marine resources the synergic combination of a number of complementary methodologies and integration of functional and informatics approaches will be required (Reen et al., 2015). The examples presented, employing a targeted and function-guided strategy, demonstrate how metagenomic technologies have advanced several research disciplines and our understanding of microbial genetic and biological diversity and ecology. Armed with information of the chemical structure and biological activity of pharmaceutically relevant compounds, an informed metagenomic strategy, in combination with *in situ* hybridization, single cell-sorting, whole genome amplification, and next generation sequencing, has successfully identified novel biosynthetic gene clusters and novel microbes that produce the metabolites. The path that led from similar compounds being found in organisms as divergent as marine sponges and beetles, to the discovery that microorganisms were the producers, and the role metagenomics played, makes a fascinating story demonstrating a perfect blend of fundamental and applied science, exemplifying the power of employing integrated technologies.

For marine metagenomics to significantly contribute to delivering pharmaceutically relevant compounds, improvements in, and integration of, various approaches and strategies is key. One of the most important hindrances encountered thus far in natural product research is re-isolation of known compounds. Thus chemical and biological de-replication is a crucial step in the process, and applies to metagenomic guided discovery as well, irrespective of the metagenomic approach employed. While sequence-based metagenomic approaches offer the power of discrimination, the expression of the pathways and the functional and biochemical characterization of the encoded products is crucial. Genome data is being produced at a dizzying pace; however, without focusing on heterologous expression challenges and the development of functional screens our capacity to uncover and develop the next generation of pharmaceutically relevant molecules will be limited (Reen et al., 2015). There are two long standing schools of thought on natural products discovery: ‘isolate and then test’ vs. ‘test and then isolate’ (Gerwick and Moore, 2012). A parallel can be drawn to employing metagenomic tools to natural product discovery: “sequence and then test” vs. “test and then sequence.” This review summarizes some of the most recent marine discoveries through the latter approach, born out of traditional chemistry-guided discovery conducted over several decades. However, to maximize our capacity to mine metagenomes for activities which have yet to be identified, parallel developments in a number of technologies need continuous attention; including biological assay screening; isolation and separation methods and analytical chemistry techniques. Peptidogenomics represents a recent advancement in high throughput mass spectrometry (MS; Kersten et al., 2011; Bouslimani et al., 2014; Medema et al., 2014). This automated approach iteratively matches the chemotypes of peptide natural products to their biosynthetic gene clusters through *de novo* tandem MS (MSn) and genome-mining (Reen et al., 2015). This constitutes a paradigm shift from the one molecule-per-study approach to drug discovery (Medema et al., 2014), and may be the key to revealing novel marine natural products from metagenomes, for advancement into the drug discovery development pipeline. There is no doubt that as yet uncultured bacteria are a rich source of novel bioactive molecules with potent therapeutic activity, and these are exciting times to be a researcher in the field.

## Conflict of Interest Statement

The authors declare that the research was conducted in the absence of any commercial or financial relationships that could be construed as a potential conflict of interest.
